# Genome-wide identification of RING finger genes in flax (*Linum usitatissimum*) and analyses of their evolution

**DOI:** 10.7717/peerj.12491

**Published:** 2021-11-15

**Authors:** Xianwen Meng, Jing Liu, Mingde Zhao

**Affiliations:** The College of Ecological Environmental and Resources, Qinghai Provincial Key Laboratory of High Value Utilization of Characteristic Economic Plants, Qinghai Tibet Alpine Wetland Restoration Engineering Technology Research Center, Qinghai Minzu University, Xining, China

**Keywords:** Flax, RING finger gene, Evolution, Expression patterns

## Abstract

**Background:**

Flax (*Linum usitatissimum*) is an important crop for its seed oil and stem fiber. Really Interesting New Gene (RING) finger genes play essential roles in growth, development, and biotic and abiotic stress responses in plants. However, little is known about these genes in flax.

**Methods:**

Here, we performed a systematic genome-wide analysis to identify RING finger genes in flax.

**Results:**

We identified 587 RING domains in 574 proteins and classified them into RING-H2 (292), RING-HCa (181), RING-HCb (23), RING-v (53), RING-C2 (31), RING-D (2), RING-S/T (3), and RING-G (2). These proteins were further divided into 45 groups according to domain organization. These genes were located in 15 chromosomes and clustered into three clades according to their phylogenetic relationships. A total of 312 segmental duplicated gene pairs were inferred from 411 RING finger genes, indicating a major contribution of segmental duplications to the RING finger gene family expansion. The non-synonymous/synonymous substitution ratio of the segmentally duplicated gene pairs was less than 1, suggesting that the gene family was under negative selection since duplication. Further, most RING genes in flax were differentially expressed during seed development or in the shoot apex. This study provides useful information for further functional analysis of RING finger genes in flax and to develop gene-derived molecular markers in flax breeding.

## Introduction

The ubiquitin-proteasome system (UPS) can degrade substrate proteins through ubiquitination, an important posttranslational modification (Smalle & Vierstra, 2004). The UPS plays essential roles in various biological processes, including growth, development, and responses to biotic and abiotic stresses in plants (Hellmann & Estelle, 2002; Kelley, 2018; Liu & Stone, 2011; Moon, Parry & Estelle, 2004; Sharma et al., 2016; Shu & Yang, 2017; Stone, 2014; Stone et al., 2005; Wang & Deng, 2011; Xu & Xue, 2019; Yu, Wu & Xie, 2016). During ubiquitination-mediated proteolysis, ubiquitin molecules are covalently attached to substrate proteins by an enzymatic cascade mediated by three enzymes: E1 ubiquitin-activating enzyme (E1s), E2 ubiquitin-conjugating enzyme (E2s), and E3 ubiquitin ligase (E3s) (Scheffner, Nuber & Huibregtse, 1995). Subsequently, the target ubiquitinated protein is degraded by the 26S proteasome. E3s usually recognize the substrate proteins and determine the specificity of ubiquitination. The large and diverse E3s in various plants could be divided into the following three types based on their structural similarities and catalytic domains: Homology to E6-AP C-Terminus (HECT), Really Interesting New Gene (RING) finger, and U-box (Kelley, 2018; Shu & Yang, 2017; Sun et al., 2019; Xu & Xue, 2019).

The RING-type ubiquitin ligase is the largest class of E3s that is defined by the presence of a RING domain composed of 40–60 amino acids (Sun et al., 2019). The RING domains are characterized by eight conserved metal ligand (ml) residues coordinating two zinc ions in a unique cross-brace structure. In this structure, ml1-ml2 and ml5-ml6 bind one zinc ion, and ml3-ml4 and ml7-ml8 bind the other (Freemont, 2000). Genome-wide analyses of RING finger genes have been reported for *Arabidopsis thaliana* (Jimenez-Lopez et al., 2018; Pavicic et al., 2017; Stone et al., 2005), *Oryza sativa* (Lim et al., 2010), *Brassica rapa* (Alam et al., 2017), *B. oleracea* (Yang & Lu, 2018), *Glycine max* (Zhang et al., 2018)*, Malus domestica* (Li et al., 2011b), *Solanum lycopersicum* (Yang et al., 2019), and *Ostreococus tauri* (Gao et al., 2016). The RING domains can be classified into two major types (RING-H2 and RING-HC) and five modified types (RING-v, RING-C2, RING-D, RING-S/T, and RING-G) based on the residues at ml positions (Stone et al., 2005).

RING finger proteins play crucial roles in various physiological processes in plants, including growth, development, and responses to abiotic and biotic stresses (Sun et al., 2019). For instance, in *A. thaliana*, DA2 and BB/EOD1 are involved in the determination of seed size or organ size (Vanhaeren et al., 2017; Xia et al., 2013), RIE1 and GW2 in seed development (Xu & Li, 2003; Zombori et al., 2020), RSL1 in seed longevity (Bueso et al., 2014), SHA1 in shoot apical meristem maintenance (Sonoda et al., 2007), SINAT5 and XBAT32 in lateral root development (Prasad et al., 2010; Xie et al., 2002), COP1 in photomorphogenesis (Xu et al., 2014), KEG in growth and development (Stone et al., 2006), NLA in adaptability to conditions involving nitrogen limitation (Peng et al., 2007), CNI1 in mediating the carbon/nitrogen response for growth phase transition (Sato et al., 2009), AtRING1A in flowering (Shen et al., 2014), DAL1/DAL2 in regulation of programmed cell death (Basnayake et al., 2011), PEX2/PEX10/PEX12 in peroxisome formation (Prestele et al., 2010; Schumann et al., 2007), SDIR1 in drought and salt responses (Zhang et al., 2007), AIRP1, RHA2a/RHA2b, XERICO, DRIP1/DRIP2, Rma1, and NERF in drought tolerance (Bu et al., 2009; Gao et al., 2015; Ko, Yang & Han, 2006; Lee et al., 2009; Li et al., 2011a; Qin et al., 2008; Ryu, Cho & Kim, 2010), STRF1 in salt responses (Tian et al., 2015), HOST1 in cold response (Dong et al., 2006), ATRF1 in aluminum tolerance (Qin et al., 2017), MIEL1 and ATL2/ATL9 in defense response (Berrocal-Lobo et al., 2010; Marino et al., 2013; Serrano & Guzmán, 2004), BRH1 in brassinosteroid responses (Molnár et al., 2002), SIS3 in sugar response (Huang et al., 2010), and DAF in anther dehiscence (Peng et al., 2013).

Flax (*Linum usitatissimum*) is an important crop that is useful for its seed oil and stem fiber (De Santana Lopes et al., 2018; Wang et al., 2012b). Although several RING finger genes have been predicted and verified in *Arabidopsis* (Jimenez-Lopez et al., 2018; Pavicic et al., 2017; Stone et al., 2005) and other plant species (Alam et al., 2017; Gao et al., 2016; Li et al., 2011b; Lim et al., 2010; Yang et al., 2019; Yang & Lu, 2018; Zhang et al., 2018), and some important transcription factors like NAC (Saha et al., 2021), WRKY (Yuan et al., 2021) and HSFs (Saha et al., 2019a) have been studied in flax, little is known about RING finger genes in flax. Therefore, in this study, we conducted a systematic genome-wide analysis to identify RING finger genes in the flax genome. Comprehensive and detailed analyses of the RING types were conducted, identifying conserved distances between ml residues, phylogenetic relationships, gene structures, domain architectures, chromosomal locations, duplication events, selection pressures, synteny, and expression patterns. This present study provides useful information for further functional analysis of the RING finger genes in flax as well as to develop gene-derived molecular markers in flax breeding.

## Materials & Methods

### Identification of RING finger proteins in *L. usitatissimum*

In order to identify all potential RING finger proteins in the flax genome, the 469, 508, and 509 RING proteins reported in *Arabidopsis* (Jimenez-Lopez et al., 2018; Pavicic et al., 2017; Stone et al., 2005) were retrieved from the TAIR database (Cheng et al., 2017), merged, and used as queries in BlastP and tBlastN (Camacho et al., 2009) against the latest *L. usitatissimum* genome database (De Santana Lopes et al., 2018; Wang et al., 2012b) in Phytozome v13 (Goodstein et al., 2012) with an *E*-value of < 10^−5^. The potential flax RING finger proteins determined from the Blast results were subsequently verified using the InterProScan5 program (Jones et al., 2014) to confirm the presence of the RING domain using the SMART (Letunic & Bork, 2017), PROSITE (Sigrist et al., 2013), and Pfam (El-Gebali et al., 2018) databases. Then, the RING domain sequences of these verified potential RING finger proteins were extracted using custom Perl scripts (File S7) and aligned using MUSCLE v3.8.31 with default parameter (Edgar, 2004) and MAFFT v7 with L-INS-i method (Rozewicki et al., 2019) to confirm the position and composition of the eight ml residues. Sequences with an incomplete RING domain or those lacking classical ml residues were excluded from the final sequence dataset.

### Phylogenetic, gene structure, and domain organization analyses of RING finger proteins

To examine the evolutionary relationships of the flax RING finger genes, the retrieved represented RING domain sequences were aligned using the MEGAX software (Kumar et al., 2018) and the MUSCLE algorithm with the default parameters. The neighbor-joining (NJ) tree of the RING domain sequences was constructed using the MEGAX software with 1,000 bootstrap repetitions. The maximum-likelihood (ML) tree of the RING domain sequences was constructed using the MEGAX software based on JTT+G+I model with 100 bootstrap repetitions. Phylogenetic trees were annotated by the ITOL v6 Web server (Letunic & Bork, 2019) and EvolView 3.0 (Subramanian et al., 2019). The gene structures of the RING finger genes were prepared using the Gene Structure Display Server 2.0 (Hu et al., 2015) through comparisons of the CDS (coding sequences) with the corresponding genomic sequences. The domain structures of the putative flax RING proteins were analyzed using SMART annotation with Genomic mode combined with the aforementioned annotation of InterProScan5 results and manually curated.

### Chromosomal locations, duplications, and microsynteny analyses of flax RING finger genes

The chromosomal locations of flax RING finger genes were obtained using BlastN with an *E*-value of < 10^−10^ against the representative genome (GCA_000224295.2) from NCBI. The MCScanX software (Wang et al., 2012a) was used to confirm duplicated and syntenic flax RING finger genes using the default settings. The chromosomal locations and microsyntenic relationships of the RING finger genes were described using the Circos−0.69-9 software (Krzywinski et al., 2009).

To show the syntenic relationships of the homologous RING finger genes in flax and other plants, syntenic analysis maps were constructed using the TBtools software (version 1.098) (Chen et al., 2020). Ks and Ka substitution rates were calculated as previously described (Khan et al., 2020). The divergence time (T, MYA) of the duplications for each paralogous gene pair was estimated by the averaged Ks values from T = Ks/2 *λ*, in which (*λ*) represents the averaged Ks substitution rate for flax, which is 1. 5 × 10^−8^ (Koch, Haubold & Mitchell-Olds, 2000; Wang et al., 2012b).

### Expression analysis of flax RING finger genes during seed developmental stages and in the AR and BR of the shoot apex

The RNA-seq data (accession number GSE130378 and GSE80178) (Li et al., 2021; Zhang & Deyholos, 2016) were downloaded from the NCBI GEO dataset (Tables S5 and S6) and filtered to explore the expression patterns of RING finger genes in flax. These transcript data (Li et al., 2021; Zhang & Deyholos, 2016) were obtained for seeds at four developmental stages (DAP5, DAP10, DAP20, and DAP30) and for the AR and BR of the shoot apex. The expression data represented log2 based Fragments per Kilobase of Exon per Million Fragments Mapped FPKM) values, and the heatmap of the expression profiles of flax RING finger genes was drawn using the heatmap.2 function in R.

## Results

### Identification of RING finger proteins in *L. usitatissimum*

In *Arabidopsis*, 469, 508, and 509 RING finger proteins have been identified or curated (Jimenez-Lopez et al., 2018; Pavicic et al., 2017; Stone et al., 2005). In the present study, using the protein sequences of merged *Arabidopsis* RING finger proteins as queries, we identified 587 RING domains (File S2) in 574 flax RING finger proteins (File S1) via Blast (Camacho et al., 2009), InterProScan (Jones et al., 2014), and domain alignment analysis (Table S1). These predicted flax RING finger proteins included 563 proteins containing only a single RING finger domain, nine containing two RING finger domains, and two containing three RING finger domains. The protein lengths in the identified flax RING finger proteins ranged from 108 AA for Lus10029456 to 4872 AA for Lus10001530. The 587 putative RING domains can be classified into seven RING types according to the amino acid residues at the eight ml locations and the distances between them: RING-H2 (292), RING-HC (204) (RING-HCa (181), RING-HCb (23)), RING-v (53), RING-C2 (31), RING-D (2), RING-S/T (3), and RING-G (2).

The 587 RING domains comprised 292 (49.74%) of the RING-H2 type, the largest domain, followed by 204 domains (34.75%) of the RING-HC type (Table 1). Based on the spacing patterns between ml7 and ml8, RING-HC was classified into two subgroups: RING-HCa (181) and RING-HCb (23) (Table 1). In addition to the two canonical RING domain types, the five modified RING domain types (RING-v, RING-C2, RING-D, RING-S/T, and RING-G) represented only 15.50% of the total identified RING domain types. RING-v represented 9.03% (53) of the total RING domains and was featured by a cysteine (Cys, C) residue at the ml4 site and a histidine (His, H) residue at the ml5 site. This pattern was reversed in RING-HC. The fourth RING type, RING-C2, represented 5.28% of the RING domains and was featured by Cys residues at the ml4 and ml5 sites, rather than the His residue observed in the RING-H2 type. In the flax genome, the three RING-S/T-type protein sequences differed from the RING-HC proteins, with a serine (Ser, S) residue at the ml2 site or ml6 site instead of a Cys residue. The two RING-D-type and the two RING-G-type proteins differed from the RING-HC proteins by an aspartic acid (Asp, D) or a glycine (Gly, G) residue at the ml5 site instead of a Cys residue.

**Table 1:** The types and features of RING domains in flax. The eight conserved metal-ligand (ml) sites in canonical and modified RING domains are shown. X(n) denotes the number of amino acids observed between the conserved ml residues.

**RING domain**		**Consensus**														
**Type**	**No.**	**ml1**		**ml2**		**ml3**		**ml4**		**ml5**		**ml6**		**ml7**		**ml8**
RING-H2	292	C	x2	C	x11-33	C	x1	H	x2	H	x2	C	x3-64	C	x2	C
RING-HCa	181	C	x2	C	x10-16	C	x1,3	H	x2,3	C	x1,2	C	x6-31	C	x1,2	C
RING-HCb	23	C	x2	C	x11-15	C	x1	H	x2,3	C	x2	C	x11-19	C	x3,4	C
RING-v	53	C	x2	C	x8-33	C	x1,2	C	x4,7	H	x2	C	x12-30	C	x2,10	C
RING-C2	31	C	x2	C	x9-15	C	x1-4	C	x2-7	C	x2	C	x1-19	C	x2	C
RING-D	2	C	x2	C	x13-15	C	x1	H	x2	D	x2	C	x10	C	x2	C
RING-S/T	3	C	x2	S	x14	C	x1	H	x2	C	x2	S	x13	C	x2	C
RING-G	2	C	x2	C	x11-16	C	x1	H	x2	G	x2	C	x12-13	C	x2	C

### Conserved spaces between ml residues in flax RING domains

**Figure 1:**
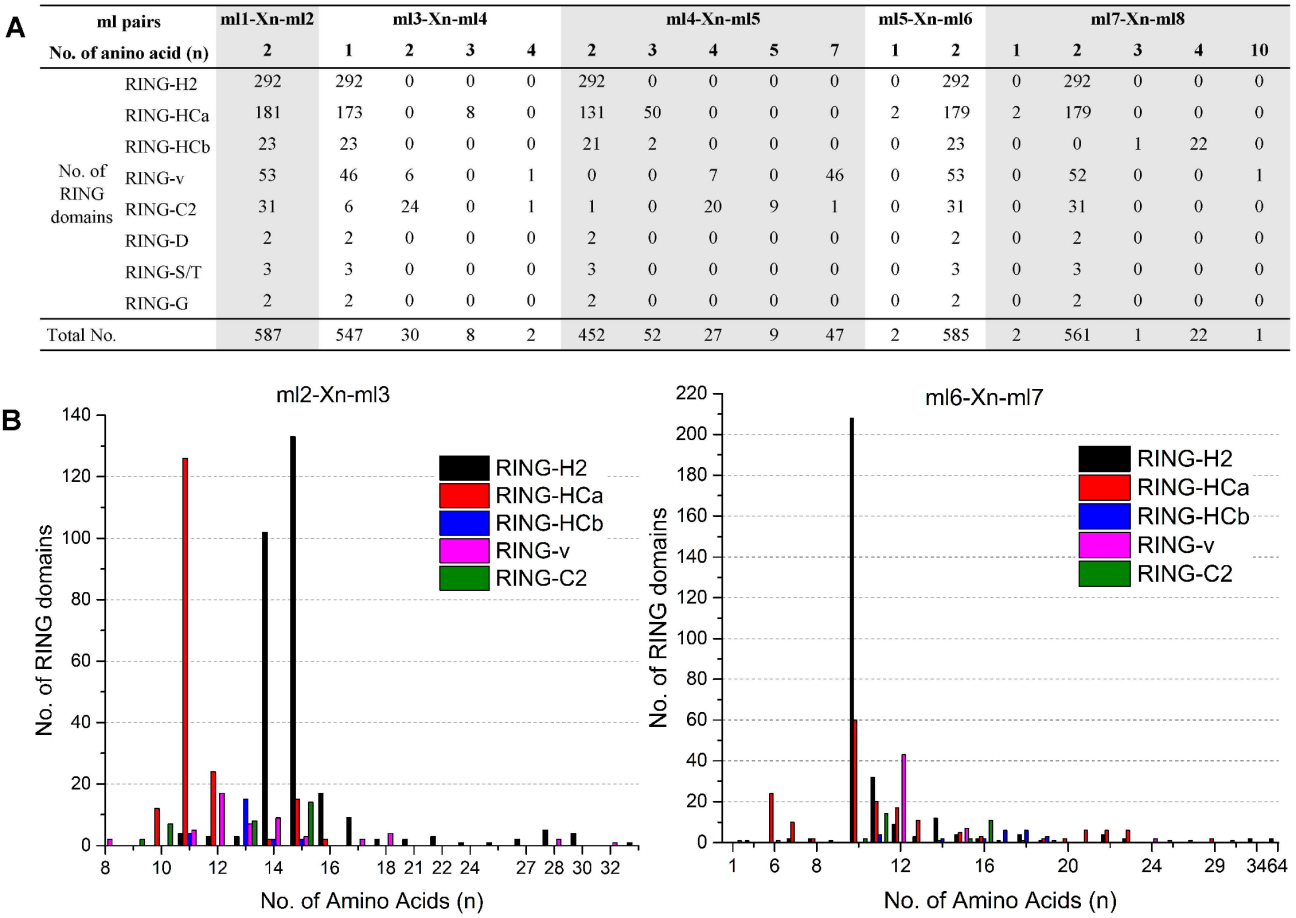
Variations in spaces between metal-ligand (ml) pairs in flax RING domains. (A) Variation in spaces between ml pairs ml1-ml2, ml3-ml4, ml4-ml5, ml5-ml6, and ml7-ml8 in different types of flax RING domains. (B) Comparison of the numbers of amino acids in the loops between ml2-ml3 and ml6-ml7 of the flax RING-H2, RING-HCa, RING-HCb, RING-v, and RING-C2 domains. X(n) denotes the number of amino acids observed between the conserved ml residues.

The eight ml residues of the RING domain coordinate two zinc ions in a unique cross-brace structure; ml1-ml2 and ml5-ml6 bind with one zinc ion, and ml3-ml4 and ml7-ml8 bind with the other (Freemont, 2000). This unique structure requires highly conserved spaces of the ml pairs ml1–ml2, ml3–ml4, ml4–ml5, ml5–ml6, and ml7–ml8, while relatively variable spaces of the ml pairs ml2-ml3 and ml6-ml7. The spaces patterns between the distinct ml residues of each of the eight putative RING types in flax are shown in Fig. 1. All 587 flax identified RING domains showed two amino acid residues (100%) between ml1 and ml2, whereas 99.66% (585/587) demonstrated two amino acid residues between ml5 and ml6, 93.19% (547/587) showed one amino acid between ml3 and ml4, 95.51% (561/587) contained two amino acids between ml7 and ml8, and 77.00% (452/587) showed two amino acid residues between ml4 and ml5 (Fig. 1A). The space between ml2 and ml3 comprised 8 to 33 residues, with 11 (140/587), 14 (118/587), and 15 (168/587) residues being most commonly observed in this space. The space between ml6 and ml7 comprised up to 64 residues, with 10 (272/587) being the most commonly observed number of residues (Fig. 1B).

Analysis of the variations in the spaces indicated patterns in the same RING types. For example, RING-H2 showed the highest number of amino acid residues between ml2 and ml3 (14 [102/292] or 15 [133/292]) sites and between ml6 and ml7 (10 [208/292]) sites. However, most of RING-HCa had only 11 (126/181) amino acid residues between ml2 and ml3 sites and 6 (24/181), 10 (60/181), 11 (20/181), or 12 (17/181) amino acids between ml6 and ml7 sites. Among RING-v, 87% showed seven amino acid residues between ml4 and ml5 sites, while 65% of RING-C2 had four amino acid residues between these sites (Fig. 1).

**Figure 2:**
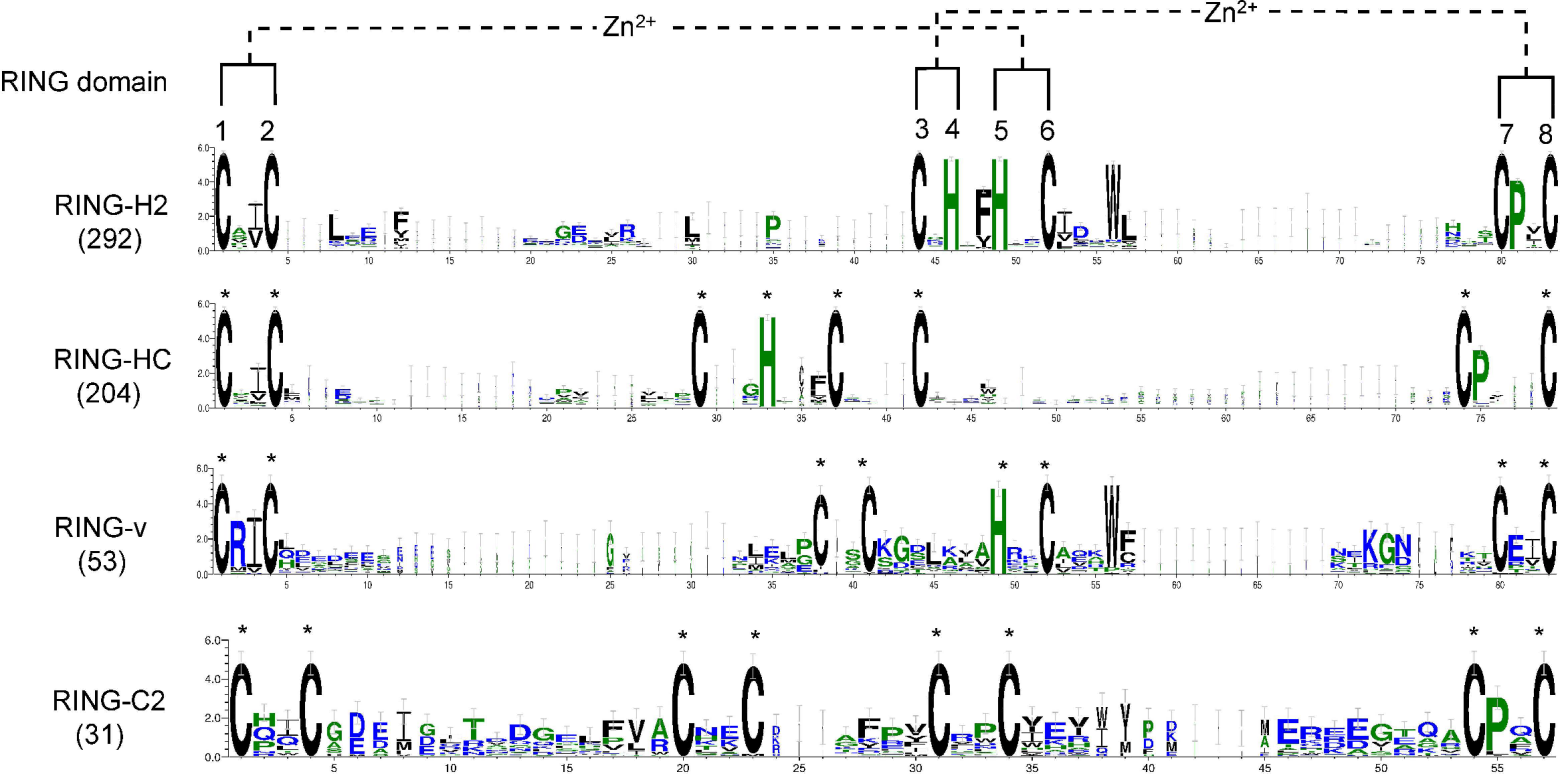
Logo of the highly conserved amino acids with metal-ligands in flax RING-H2, RING-HC, RING-v, and RING-C2 domains. The eight conserved metal-ligand residues are marked with asterisks, and zinc-coordinating amino acid pairs are shown. Bits in the *Y*-axis represent the amount of informational content at each sequence position.

To examine whether any amino acid residues other than those involved in ml pairing are highly conserved in the flax RING domain sequences, a multiple sequence alignment of all RING domain sequences was conducted, and domain sequence logos of the distinct representative RING types were performed (Fig. 2). Ile (I) or Val (V) was the most frequently observed amino acid residue ahead of ml2 in the distinct RING types. Another highly conserved amino acid was Pro (P), which was always observed at the second site after ml7 site in over 94% of the putative RING domains, except RING-v, which showed an E amino acid instead. In the RING-H2 type, a phenylalanine (Phe, F) most often preceded the ml5 site, a leucine (Leu, L) was often present next to the ml2 site, and a D was typically present at the second site after the ml6 site. A G regularly existed in front of the ml4 site in RING-HC. More than 88% of RING-H2 and RING-v included a tryptophan (Trp, W) at the fourth site after the ml6 site. The residue after the ml1 site in RING-v was almost often an arginine (Arg, R).

### Domain organization in flax RING finger proteins

To better classify the flax RING finger proteins, full sequences of the 574 identified RING finger proteins were examined using the SMART database and InterProScan to verify the conserved domain organization. Besides the RING domain, 68 additional domains were identified in these proteins, and based on their organization, they were classified into 45 groups and corresponding subgroups (Table S2). Group 1 (without additional domains, included 188 members) and group 2 (one or more transmembrane domains, included 157 members) were the two largest groups, accounting for 60% of the total proteins. The other groups or subgroups usually contained fewer members, and most of them had only several members. These additional domains in the putative flax RING finger proteins were predicted to play essential functional roles. The predicted protein-binding domains might be related to substrate recognition, including ANK, BRCT, SPRY, Vwaint, and WD40. The domains participating in ubiquitination involved CUE, GIDE, and RWD. The predicted nucleic acid-binding domains involved DEXDc, HIRAN, RRM, ZnF-NFX, ZnF-C2H2, and ZnF-C3H1. In this research, several domains, for examples Zn ^2+^ binding domains (ZnF-CHY, ZnF_NFX, ZnF_RBZ, Zinc_ribbon_6, and Zinc_ribbon_9) and heavy metal ion binding domains (HMA), were predicted to be involved in binding metal ions. The previously verified domains such as IBR, PA, Pep3_Vps18, SPRY, Zinc_Ribbon_6, Zinc_Ribbon_9, DUF1117, DUF1232, and DUF3675, were found to be associated with RING domains. Most of these additional domains were observed in diverse plants such as *A. thaliana, O. sativa, V. vinifera,* and *P. trichocarpa*, indicating that their functions could be conserved, and they may perform the same or similar functions among these species.

### Phylogenetic and structural analyses of flax RING finger genes

**Figure 3:**
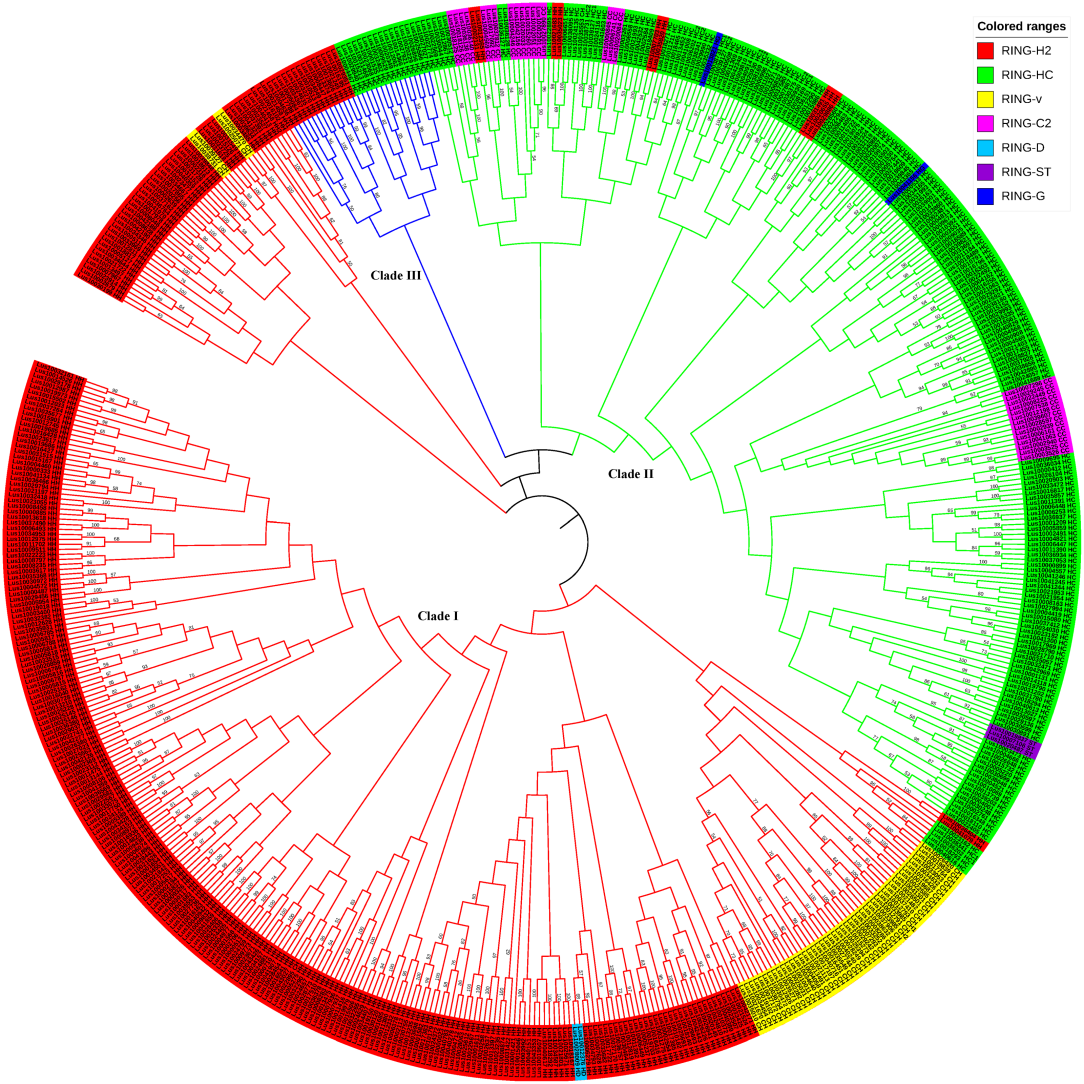
Phylogenetic relationships of RING finger genes in flax. The neighbor-joining (NJ) tree constructed from 587 RING domains observed in 574 RING finger proteins in flax is shown. The colored ranges corresponding to the RING types in flax are shown. The three main branches, Clade I, II, and III, are represented in red, green, and blue color, respectively.

To examine the evolutionary relationships among the RING genes in flax, the 587 RING domain sequences were aligned using the MEGAX software employing the MUSCLE algorithm with default parameters to construct a neighbor-joining (NJ) phylogenetic tree with 1,000 bootstrap repetitions (Fig. 3, Figs. S1, S2, and File S2) and a ML tree based on JTT+G+I model with 100 bootstrap repetitions (Fig. S4 and File S6). Based on the evolutionary analysis, the flax RING genes could be classified into three clades: Clade I (RING-H2 Clade), Clade II (RING-HC Clade), and Clade III (RING-temp Clade). Clade I comprised the highest number of RING-H2, RING-v, and RING-D genes and several RING-HC genes. Clade II included the highest number of RING-HC, RING-C2, RING-G, and RING-ST genes and several RING-H2 genes. Clade III consisted of RING-H2 and RING-HC genes.

### Chromosome locations and duplication of flax RING finger genes

**Figure 4:**
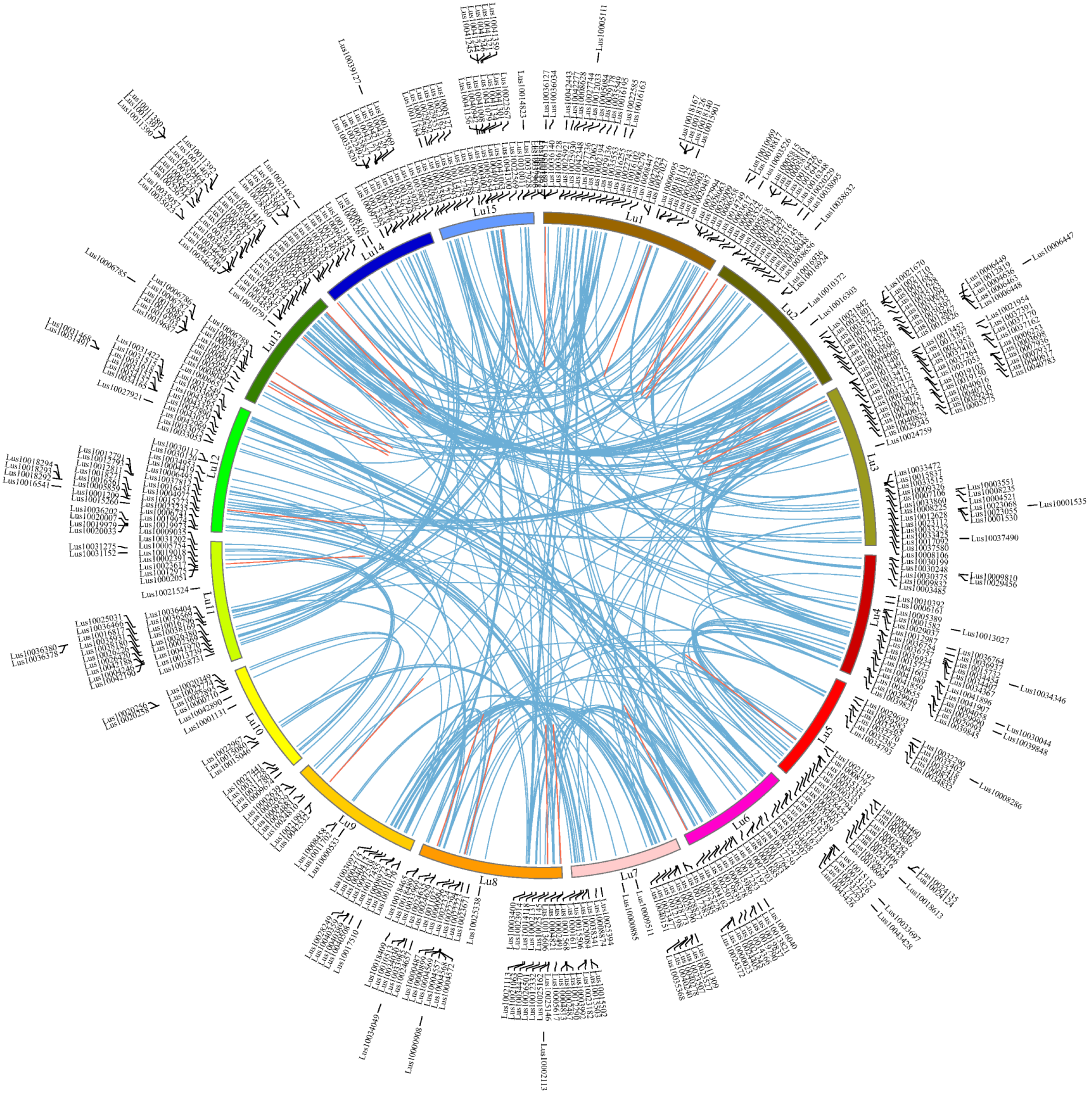
Chromosome locations of RING finger genes and duplicated gene pairs in the flax genome. Chromosomes 1–15 are shown with different colors and in a circular form. The approximate distribution of each flax RING finger gene is marked on the circle with a short black line. Colored curves denote the details of syntenic regions between flax RING finger genes (blue and red curves represent the segmental duplication events and the tandem duplication events, respectively).

To investigate the chromosome locations of the predicted 574 RING finger genes in flax, the DNA sequences of all flax genes in Phytozome v13 were queried using BlastN against the representative genome assemble (GCA_000224295.2, chromosome level) from NCBI (*E*-value <10^−10^). The chromosomal locations of the 574 RING genes (Files S4 and File S5) were extracted from the Blast results under strict control. All the RING finger genes were distributed among 15 flax chromosomes at different densities: Lu1-Lu15 comprised 54, 47, 56, 39, 34, 35, 27, 45, 23, 15, 32, 47, 49, 34, and 37 RING finger genes, respectively (mean: 38; max: Lu3, 56; min: Lu10, 15) (Fig. 4). To examine the gene duplication events, all the flax RING finger genes were analyzed using BlastP and the MCScanX software. A total of 312 segmental duplication events and 27 tandem duplication events were confirmed in the whole flax genome (Table S3). To examine the selection pressure on the flax RING finger genes, the synonymous (Ks) and nonsynonymous (Ka) substitution rates and the Ka/Ks ratio of the RING finger gene pairs were calculated (Table S3). The Ka/Ks ratio was estimated to be less than 1, indicating that the duplicated RING finger genes in flax were under strong negative selection. Gene duplication analysis indicated that both segmental and tandem duplication events contributed to the expansion of the flax RING finger gene family.

**Figure 5:**
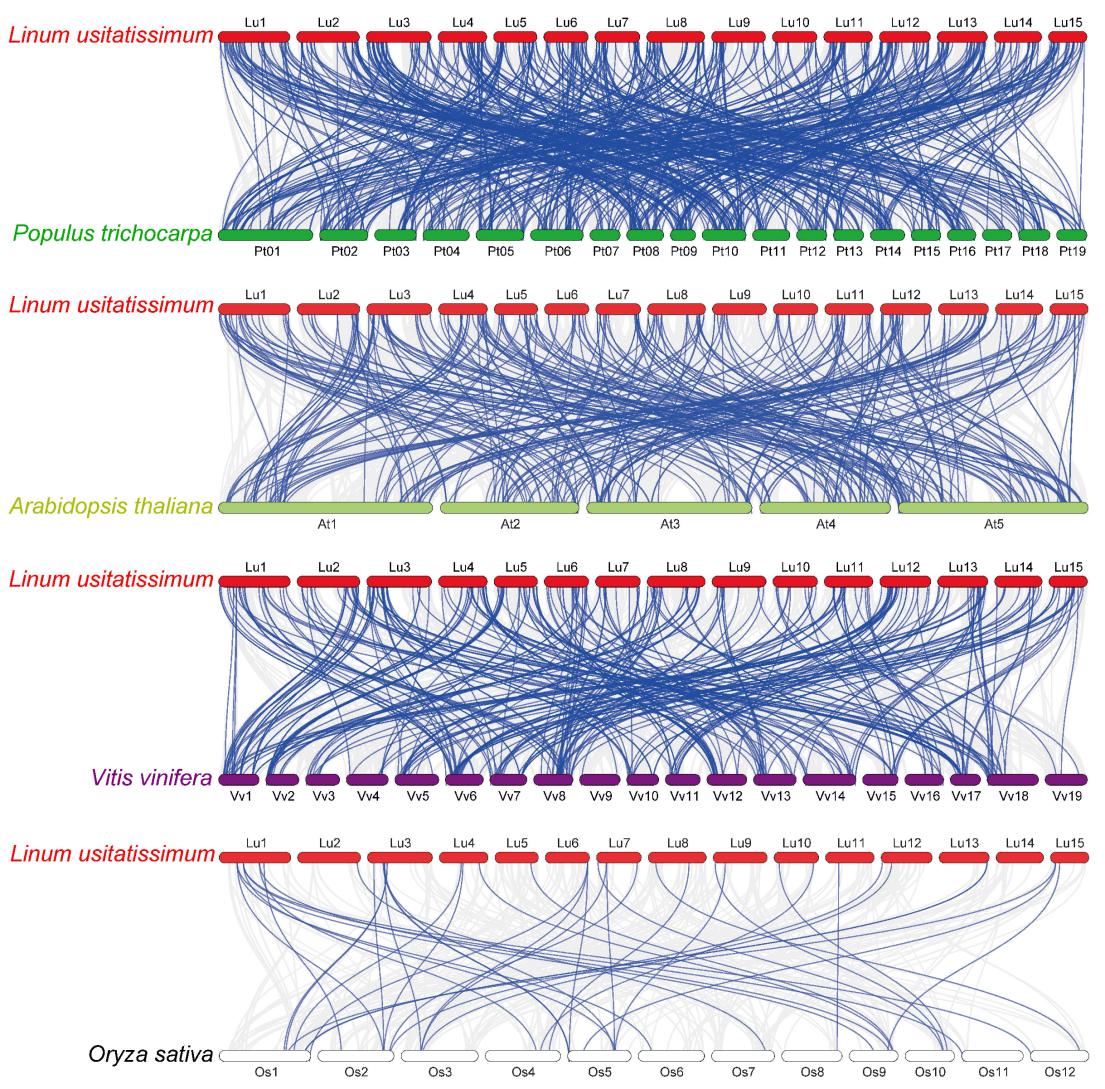
Synteny analysis of RING finger genes in flax and four representative plant species. Gray lines in the background denote collinear blocks within flax and other plant genomes, and blue lines highlight the syntenic RING gene pairs, with four representative plant species being *P. trichocarpa, A. thaliana, V. vinifera,* and *O. sativa*.

To investigate the phylogenetic mechanisms of the flax RING finger genes, a comparative syntenic analysis (Fig. 5) of flax and the other four representative plants, including three dicots (*P. trichocarpa*, *A. thaliana*, and *V. vinifera*) and one monocot (*O. sativa*), was performed. A total of 572, 304, 314, and 42 orthologous RING finger gene pairs were observed between flax and the other four species (*P. trichocarpa, A. thaliana, V. vinifera,* and *O. sativa*), respectively. Additionally, most of the orthologous RING finger genes demonstrated a Ka/Ks ratio of less than 1, indicating that the RING finger gene family was under a strong negative selection pressure during the evolution process (Table S4).

**Figure 6:**
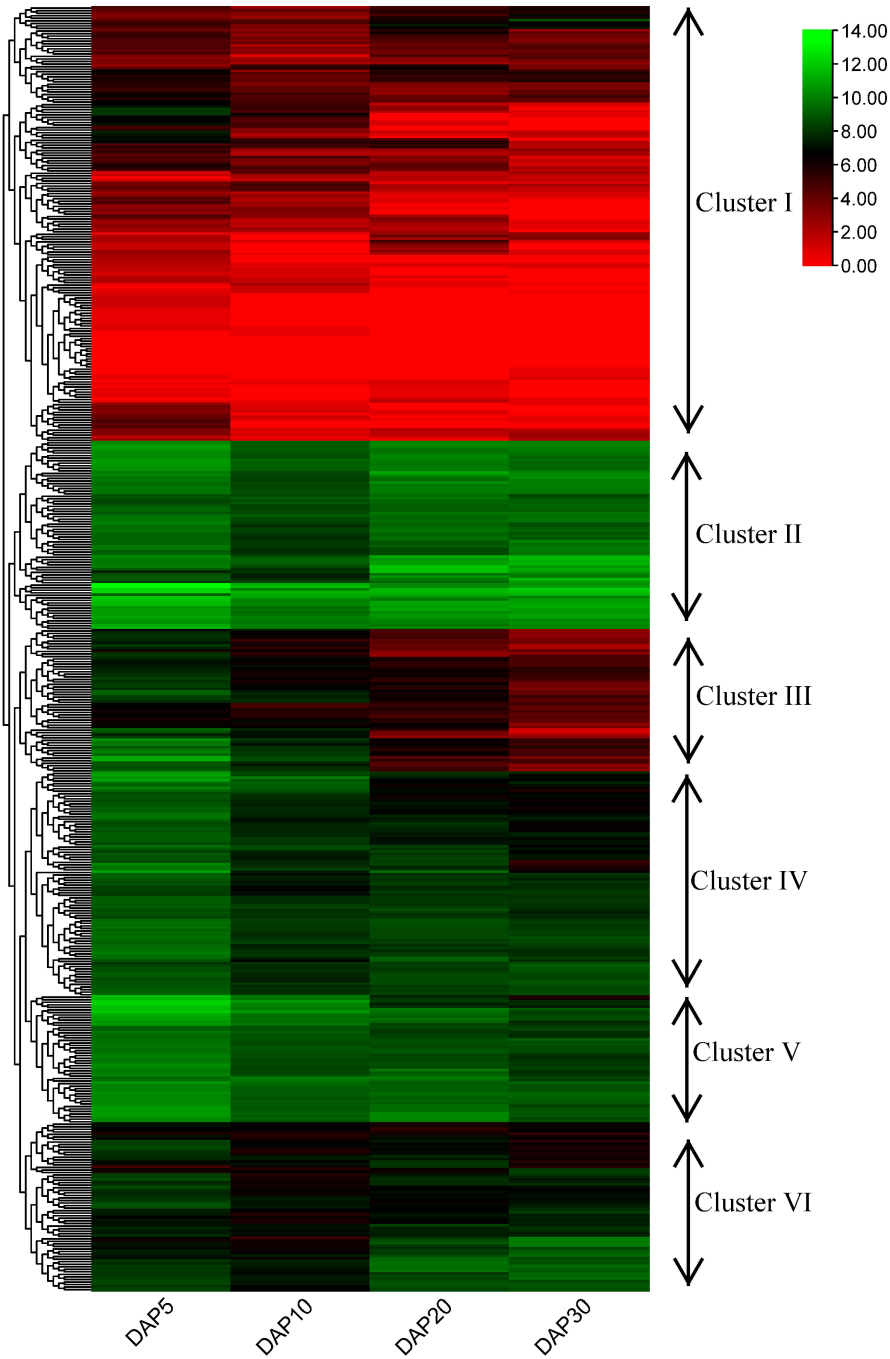
Heatmap and hierarchical clustering of expression of RING finger genes during the development of flax seeds.

### Expression of flax RING finger genes during seed developmental stages

To examine the potential functions of RING finger genes during the seed development process in flax, a published flax RNA sequencing (RNA-seq) dataset (NCBI GEO accession: GSE130378) (Li et al., 2021) was used to confirm the expression profiles of these genes. A total of 505 RING finger genes were expressed at four stages (days after pollination [DAP]5, DAP10, DAP20, and DAP30) during the seed development process in flax, determined based on the transcriptome data; the other 69 genes were not found and excluded from the analysis (Fig. 6, Table S5). According to the hierarchical clustering of the expression patterns, these 505 genes were classified into six clusters. The RING finger genes in Cluster I (including 171 members) exhibited relatively lower expression levels at most stages, whereas *Lus10023055*, *Lus10041156*, *Lus10040617*, and *Lus10025145*, and *Lus10012819* showed relatively higher expression at a specific stage than other genes in Cluster I. In Cluster II (including 74 members), typically, genes were highly expressed at different stages. For instance, *Lus10007538* and *Lus10028597* showed stronger expression than other clusters. *Lus10011416* and *Lus10009695* showed less abundance in seeds at DAP5 or DAP10. In Cluster III, IV, and V (including 56, 88, and 50 members), with an increase in the number of days, gene expression showed a downward trend, whereas the expression of genes in Cluster III and IV seemed to decrease faster than that of those in Cluster V. Additionally, genes in Cluster V showed relatively higher expression than those in Cluster III and IV at the same stages. The expression pattern of genes in Cluster VI (including 66 members) was different from that of others. The expression levels remained high at DAP5, decreased at DAP10, and increased again at DAP20 or DAP30.

**Figure 7:**
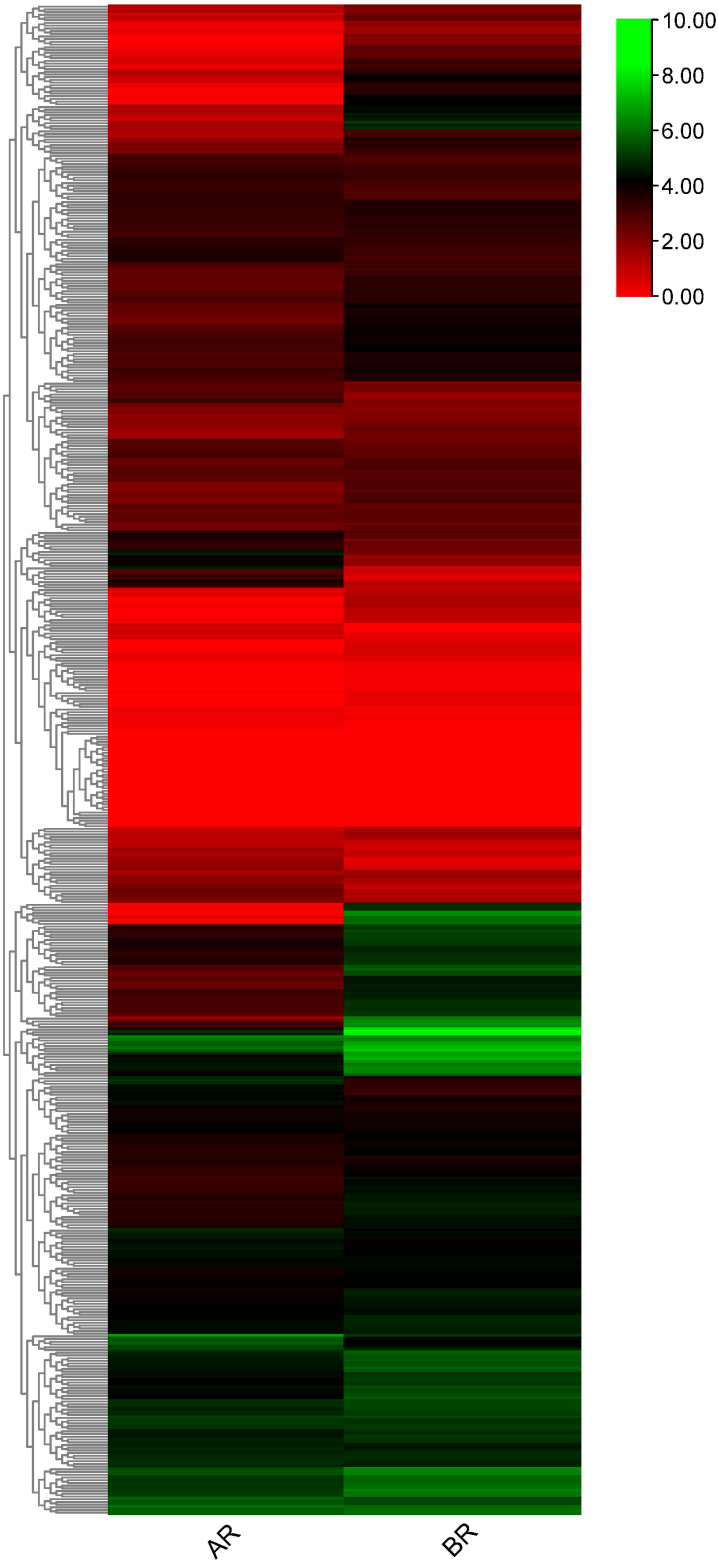
Heatmap generated for flax RING finger genes derived from RNA sequencing data (accession no. GSE80718) using log2 transformed average Fragments per Kilobase of Exon per Million Fragments Mapped (FPKM) values.

### Differential expression patterns of flax RING finger genes in the apical region (AR) and basal region (BR) of the shoot apex

To investigate RING finger genes that may contribute to the specification of phloem fiber identity in flax, publicly available RNA-seq data (NCBI GEO accession: GSE80718) (Zhang & Deyholos, 2016) were used to determine the differential expression patterns of these genes. 556 RING finger genes were expressed in the differentially expressed transcriptome data of the flax shoot apex. A total of 216 genes showed differential expression patterns in the apical and basal tissues (Fig. 7, Table S6). A total of 49 genes, including *Lus10040716* and *Lus10002639*, showed high expression in the AR and showed low expression in the BR, whereas the other 167 genes, including *Lus10013210*, *Lus10012811*, and *Lus10030728*, demonstrated low expression in the AR and high expression in the BR. Differential transcript expression data corresponding to the shoot apex in which fiber specification is observed would complement other approaches aimed at understanding primary phloem fiber differentiation.

## Discussion

In this study, we performed a comprehensive analysis of flax RING finger genes, including the RING types, conserved spaces between ml pairs, phylogenetic relationships, gene structures, domain architectures, chromosomal locations, duplication events, selection pressures, synteny, and expression patterns.

We identified 574 putative RING finger proteins in the flax genome. The number of RING finger genes in flax was higher than that reported for *A. thaliana* (469), *O. sativa* (425), or *S. lycopersicum* (469), and lower than that reported for *B. rapa* (715) or *B. oleracea* (735). However, the RING finger genes accounted for approximately 1.3% of the putative protein-encoding genes in flax, similar to the proportions reported for *O. sativa* (1.2%), *S. lycopersicum* (1.3%), and *B. rapa* (1.5%) but were lower than those reported for *A. thaliana* (approximately 2%). A possible explanation for this phenomenon involves genome duplication, which may have contributed to the increase in the number of RING finger genes in *A. thaliana* and other plant species, such as flax and *B. rapa*. During the evolution process, under certain specific selection pressures, several RING finger genes were lost in the genome or fixed by neofunctionalization or subfunctionalization. Based on this perspective, we speculate that the RING finger genes may reflect the trails in sequences or functions during the plant evolution.

The common features verified in the 587 RING domains in 574 putative RING finger proteins in flax were similar to those observed in previous studies, including RING types, conserved spacing patterns between ml residues, conserved residues, and domain architecture. Based on phylogenetic relationships, the flax RING finger genes could be classified into Clade I (RING-H2), Clade II (RING-HC), and Clade III (RING-temp). These three phylogenetic clades aid in understanding the evolution of RING finger genes in flax. Based on this, we can speculate that the RING-v genes were possibly derived from an ancestor common with the RING-H2 clade, and RING-C2 genes may have been derived from an ancestor common with the RING-HC clade. Interestingly, RING-D and RING-G genes differ only with respect to the fifth ml pair of the eight conserved ml residues, but they may originate from different clades based on current results. Clade III, the temporary RING clade, consisting of RING-H2 genes and RING-HC genes, might be an intermediate evolution state of the RING finger genes in plants. Based on these results, it is difficult to speculate whether the common ancestor was RING-H2 or RING-HC. Hence, further investigations of RING finger genes in multiple plant species are required. The RING finger genes of each group or subgroup often have same or similar gene structures and domain organizations (Table S2, Fig. S3), suggesting that they probably target, bind, or interact with identical or similar substrate proteins. The presence of the diverse groups of RING finger proteins may be attributed to various roles of the RING-type E3 ligases, which are involved in plant growth, development, and responses to environmental stresses.

Gene duplications, including segmental duplication and tandem duplication, are important evolutionary mechanisms accelerating the rapid expansion of gene families (Panchy, Lehti-Shiu & Shiu, 2016). Segmental duplication events are frequently observed in most plant genomes, as most plant species present are diploidized polyploids, and their present genomes maintain abundant duplicated chromosomal blocks (Zhu et al., 2014). In the present study, over 80% of RING finger genes (463/574) were characterized as duplicated genes in the flax genome (Fig. 4), generating 312 segmental duplications and 27 tandem duplications. This observation indicates that segmental duplications clearly contributed to the expansion of the flax RING finger genes. The flax genome underwent two polyploidization events that contributed to the shaping of the present genome, namely, one mesopolyploidization event at 3.7-9 MYA and one palaeopolyploidization event at 20-44 MYA (Sveinsson et al., 2014; Wang et al., 2012b; You et al., 2018). By calculating the divergence intervals of the duplicated RING finger gene pairs in the flax genome, we inferred that 200 duplicated pairs (average divergence time: 4.76 million years ago [MYA]) of the flax RING finger paralogous genes derived from a recent duplication, and 132 duplicated pairs (average divergence time: 35.38 MYA) of the flax RING finger paralogous genes derived from an ancient duplication during the shaping of the flax genome (Table S3). Thus, segmental duplications were the main driving forces behind the evolution of RING finger genes during speciation and functionalization.

To further understand the functions of RING finger genes in seed development and the different shoot apex regions of flax, we performed expression analysis of publicly available RNA-seq data. We found that most of the RING finger genes showed distinct expression levels (Figs. 6 and 7). At different flax seed developmental stages, approximately 66% of the RING finger genes showed high expression and exhibited six different expression patterns (Fig. 6). This finding indicates that these RING finger genes may be involved in flax seed development and were perhaps associated with seed size and production or oil accumulation and quality. The expression profiles of the two important regions in the shoot apex region of flax showed differential expression patterns of RING finger genes (Fig. 7), suggesting that RING finger genes may be involved in the development of the shoot apex, and the differentiation directions of different tissues might be regulated by different RING finger genes. Further studies examining the functions of these RING finger genes in flax would provide more insights into the regulatory mechanisms of these genes in plant growth, development, and response to stresses. The various large potential functional members of RING finger genes may be used for developing RING finger gene-derived molecular markers in flax breeding (Saha et al., 2019b). For example, there are several drought resistance-related genes, such as *AIRP1*, *RHA2a/RHA2b*, *XERICO*, *DRIP1/DRIP2*, *Rma1*, and *NERF* (Bu et al., 2009; Gao et al., 2015; Ko, Yang & Han, 2006; Lee et al., 2009; Li et al., 2011a; Qin et al., 2008; Ryu, Cho & Kim, 2010), in RING finger genes, which may be used as important indices for screening high drought-resistant flax varieties. This information will provide an important direction for the verification of flax breeding experiments in our future studies.

## Conclusions

In this study, 574 flax RING finger genes were identified and their characteristics, including the RING types, conserved spaces between ml pairs, phylogenetic relationships, gene structures, domain architectures, chromosomal locations, duplication events, selection pressures, synteny, and expression patterns were studied. These RING finger genes were unevenly distributed among 15 flax chromosomes. A total of 312 inferred segmental duplicated gene pairs from 411 RING finger genes indicated that segmental duplication events contributed greatly to the expansion of the flax RING finger gene family. The expression analysis of publicly available RNA-seq data revealed that these RING finger genes may be involved in the development of flax seed and the shoot apex. Our comprehensive analysis of flax RING finger genes will inform future studies examining the functions of RING finger genes and developing gene-derived molecular markers in flax breeding.

##  Supplemental Information

10.7717/peerj.12491/supp-1 Supplemental Information 1 Phylogenetic relationships of RING finger genes in flax (unrooted tree) Phylogenetic relationships of RING finger genes in flax (unrooted tree). The neighbor joining (NJ) tree constructed from 587 RING domains in 574 RING finger proteins in flax is shown. The colored ranges corresponding to the RING types in flax are indicated with different colors. MEGAX package was used to construct the NJ tree from domain sequence alignments (File S2) of flax RING finger genes, with 1000 bootstrap replicates. Numbers refer to bootstrap support in terms of percentage.

10.7717/peerj.12491/supp-2 Supplemental Information 2 Phylogenetic relationships of RING finger genes in flax (curved tree) The neighbor joining (NJ) tree constructed from 587 RING domains in 574 RING finger proteins in flax is shown. The colored ranges corresponding to the RING types in flax are indicated with different colors. MEGAX package was used to construct the NJ tree from domain sequence alignments (File S2) of flax RING finger genes, with 1000 bootstrap replicates. Numbers refer to bootstrap support in terms of percentage.

10.7717/peerj.12491/supp-3 Supplemental Information 3 Gene structure of RING finger genes (tree order) in flax

10.7717/peerj.12491/supp-4 Supplemental Information 4 Phylogenetic relationships of RING finger genes in flax (ML tree) The maximum-likelihood (ML) tree constructed from 587 RING domains in 574 RING finger proteins in flax is shown. MEGAX package was used to construct the ML tree from domain sequence alignments (File S2) of flax RING finger genes, based on JTT+G+I model with 100 bootstrap replicates. Numbers refer to bootstrap support in terms of percentage.

10.7717/peerj.12491/supp-5 Supplemental Information 5 FASTA format of the 574 full-length RING finger protein sequences in flax genome

10.7717/peerj.12491/supp-6 Supplemental Information 6 FASTA format multiple sequence alignments of 587 RING finger domain sequences in flax genome

10.7717/peerj.12491/supp-7 Supplemental Information 7 NJ phylogenetic tree of 587 RING finger domain sequences in flax genome (Newick format) The neighbor-joining (NJ) tree of the RING domain sequences was constructed using the MEGAX software with 1,000 bootstrap repetitions.

10.7717/peerj.12491/supp-8 Supplemental Information 8 FASTA format of the 574 RING finger coding sequences in flax genome

10.7717/peerj.12491/supp-9 Supplemental Information 9 Chromosome locations of 574 RING finger genes in flax genome

10.7717/peerj.12491/supp-10 Supplemental Information 10 ML phylogenetic tree of 587 RING finger domain sequences in flax genome (Newick format) The maximum-likelihood (ML) tree of the RING domain sequences was constructed using the MEGAX software based on JTT+G+I model with 100 bootstrap repetitions.

10.7717/peerj.12491/supp-11 Supplemental Information 11 The custom perl script for domain sequence extraction

10.7717/peerj.12491/supp-12 Supplemental Information 12 Information of 574 RING finger genes in flax

10.7717/peerj.12491/supp-13 Supplemental Information 13 Flax RING finger domain containing protein groups based on the presence and organization of additional domain(s)

10.7717/peerj.12491/supp-14 Supplemental Information 14 Duplication events in flax RING finger genes

10.7717/peerj.12491/supp-15 Supplemental Information 15 Orthologous relationships of RING finger genes between flax and other four plant species

10.7717/peerj.12491/supp-16 Supplemental Information 16 FPKM transcription count data for 505 flax RING finger genes during seed developmental stages (DAP5, DAP10, DAP20, and DAP30; DAP: days after pollination)

10.7717/peerj.12491/supp-17 Supplemental Information 17 FPKM transcription count data for 556 flax RING finger genes in the apical region (AR) and the basal region (BR) of the shoot apex

## Additional Information and Declarations

### Data Availability

The following information was supplied regarding data availability: The data is available at Phytozome v13: - Arabidopsis thaliana Araport11 (https://phytozome-next.jgi.doe.gov/info/Athaliana_Araport11); - Linum usitatissimum v1.0 (https://phytozome-next.jgi.doe.gov/info/Lusitatissimum_v1_0); - Oryza sativa v7.0 (https://phytozome-next.jgi.doe.gov/info/Osativa_v7_0); - Populus trichocarpa v4.1 (https://phytozome-next.jgi.doe.gov/info/Ptrichocarpa_v4_1); - Vitis vinifera v2.1 (https://phytozome-next.jgi.doe.gov/info/Vvinifera_v2_1); The representative chromosome-level flax genome is available at NCBI Genome: GCA_000224295.2. The RNA-seq data of flax is available at NCBI GEO: GSE130378, GSE80178.
