# Supplementary figures and images for: Genome-wide identification of RING finger genes in flax (Linum usitatissimum) and analyses of their evolution
Source: PeerJ. 2021 Nov 15;9:e12491. doi: 10.7717/peerj.12491 (PMC8601054; doi:10.7717/peerj.12491)

Tree scale:

**Colored ranges**

- RING-H2
- RING-HC
- RING-v
- RING-C2
- RING-D
- RING-ST
- RING-G

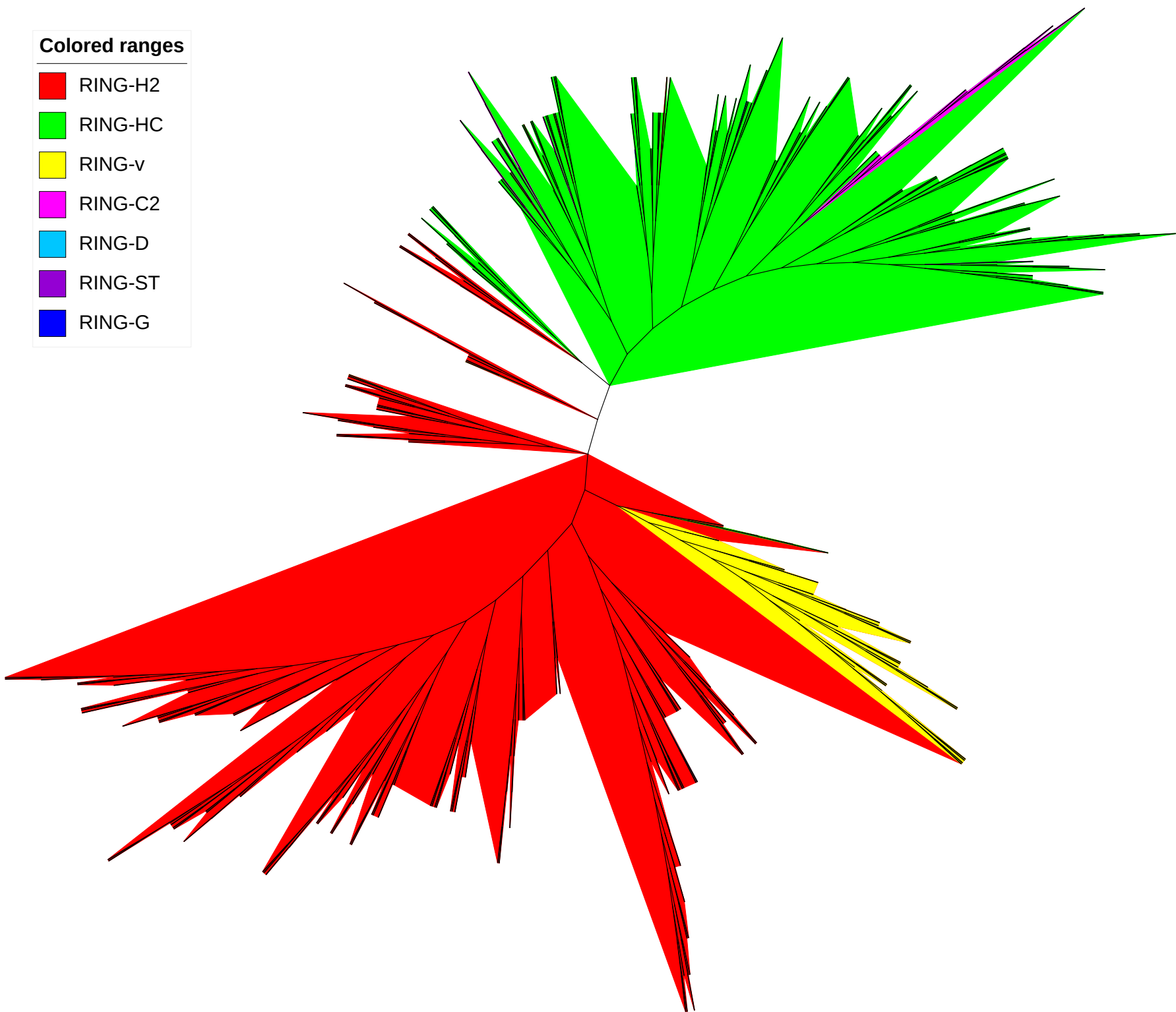

Supplement: Supplemental Information 1 — Phylogenetic relationships of RING finger genes in flax (unrooted tree). The neighbor joining (NJ) tree constructed from 587 RING domains in 574 RING finger proteins in flax is shown. The colored ranges corresponding to the RING types in flax are indicated with different colors. MEGAX package was used to construct the NJ tree from domain sequence alignments (File S2) of flax RING finger genes, with 1000 bootstrap replicates. Numbers refer to bootstrap support in terms of percentage. [file peerj-09-12491-s001.pdf]

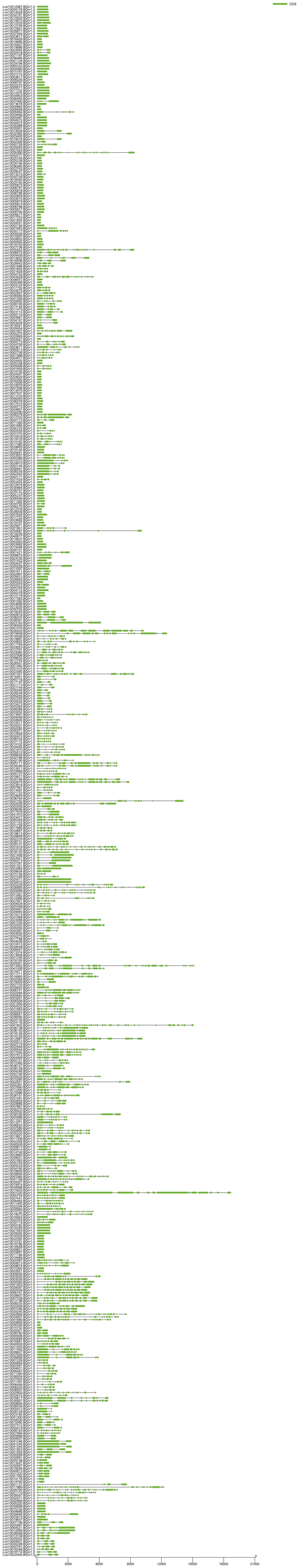

Supplement: Supplemental Information 3 [file peerj-09-12491-s003.pdf]

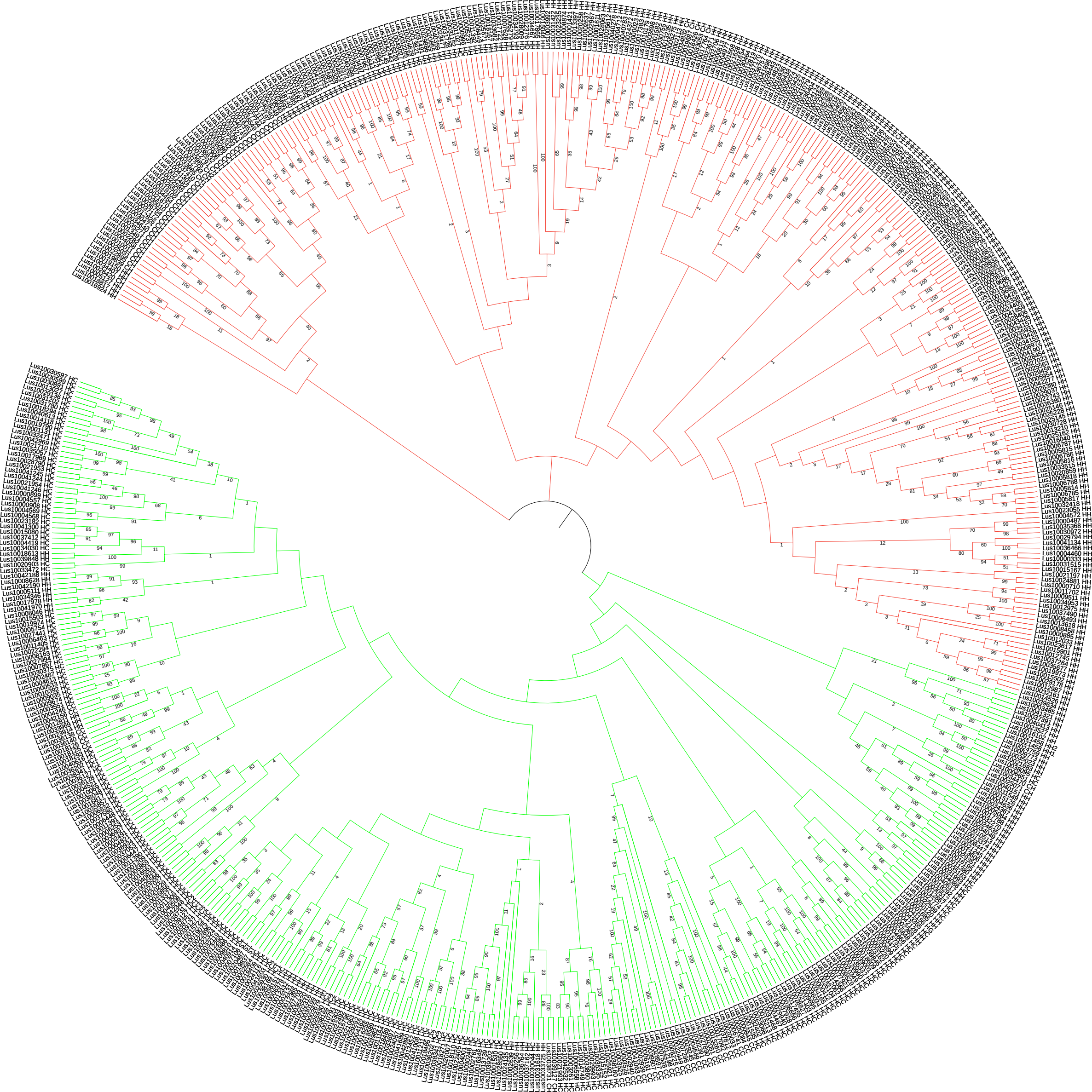

Supplement: Supplemental Information 4 — The maximum-likelihood (ML) tree constructed from 587 RING domains in 574 RING finger proteins in flax is shown. MEGAX package was used to construct the ML tree from domain sequence alignments (File S2) of flax RING finger genes, based on JTT+G+I model with 100 bootstrap replicates. Numbers refer to bootstrap support in terms of percentage. [file peerj-09-12491-s004.pdf]
