# Supplementary material for: Genome-wide identification of RING finger genes in flax (Linum usitatissimum) and analyses of their evolution
Source: PeerJ. 2021 Nov 15;9:e12491. doi: 10.7717/peerj.12491 (PMC8601054; doi:10.7717/peerj.12491)
Supplement: Supplemental Information 2 — The neighbor joining (NJ) tree constructed from 587 RING domains in 574 RING finger proteins in flax is shown. The colored ranges corresponding to the RING types in flax are indicated with different colors. MEGAX package was used to construct the NJ tree from domain sequence alignments (File S2) of flax RING finger genes, with 1000 bootstrap replicates. Numbers refer to bootstrap support in terms of percentage. [file peerj-09-12491-s002.pdf]

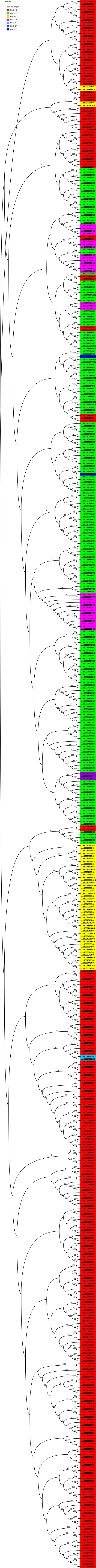
Tree scale:
Colored ranges
RING-H2
RING-HC
RING-v
RING-C2
RING-D
RING-ST
RING-G
